# Drug–Target Interaction Prediction Based on an Interactive Inference Network

**DOI:** 10.3390/ijms25147753

**Published:** 2024-07-15

**Authors:** Yuqi Chen, Xiaomin Liang, Wei Du, Yanchun Liang, Garry Wong, Liang Chen

**Affiliations:** 1 College of Mathematics and Computer, Shantou University, Shantou 515063, China; 21yqchen2@stu.edu.cn (Y.C.); 19xmliang@stu.edu.cn (X.L.); 2 Key Laboratory of Symbol Computation and Knowledge Engineering of Ministry of Education, College of Computer Science and Technology, Jilin University, Changchun 130012, China; weidu@jlu.edu.cn (W.D.); ycliang@jlu.edu.cn (Y.L.); 3 Faculty of Health Sciences, University of Macau, Taipa, Macau SAR 999078, China; garrygwong@um.edu.mo

**Keywords:** DTI, interactive inference network, convolutional neural network, self-attention, drug repurposing

## Abstract

Drug–target interactions underlie the actions of chemical substances in medicine. Moreover, drug repurposing can expand use profiles while reducing costs and development time by exploiting potential multi-functional pharmacological properties based upon additional target interactions. Nonetheless, drug repurposing relies on the accurate identification and validation of drug–target interactions (DTIs). In this study, a novel drug–target interaction prediction model was developed. The model, based on an interactive inference network, contains embedding, encoding, interaction, feature extraction, and output layers. In addition, this study used Morgan and PubChem molecular fingerprints as additional information for drug encoding. The interaction layer in our model simulates the drug–target interaction process, which assists in understanding the interaction by representing the interaction space. Our method achieves high levels of predictive performance, as well as interpretability of drug–target interactions. Additionally, we predicted and validated 22 Alzheimer’s disease-related targets, suggesting our model is robust and effective and thus may be beneficial for drug repurposing.

## 1. Introduction

The process of drug discovery is complex and full of challenges. The process must identify target molecules, utilize and select efficient screening methods, search for lead compounds, optimize leads, navigate and successfully pass clinical trials, and if these barriers are overcome, continue with post-market monitoring. Each step requires considerable resources and is time-consuming. If drugs pass these barriers and enter into clinical use, additional uses and benefits should be explored. Most drugs do not interact with a single target molecule, which could result in unexpected therapeutic effects [1]. This fortuitous event could potentially help to discover a new indication for an existing drug due to its multiple sites of action. In order to exploit this possibility, drug–target interaction (DTI) prediction has been developed and is the basis for identifying new drugs and new indications based upon existing drugs in use (drug repurposing) [2].

Identifying new drugs and their targets is still extremely difficult due to the complex relationship between chemical and genome space. Drugs and their targets interact due to various factors, including chemical bonds related to their affinity [3]. Virtual screening for drug–target interactions provides a better understanding of complex biological interactions and essential biological processes and identifies new potential drug–target interaction candidates for subsequent experimentation and validation. To assist virtual screening, there are generally three types of approaches: ligand-based approaches [4], molecular docking-based approaches [5], and chemical genomics-based approaches [6]. Ligand-based approaches involve comparing and analyzing the degree of similarity between candidate compounds and target ligands. However, at present, the number of known ligands is limited. In molecular docking-based approaches, the three-dimensional structure of the drug and target is examined to identify potential binding sites, which is time-consuming and computationally expensive. The chemical genomics-based approaches successfully break through the limitations by taking advantage of the genomic information of targets and chemical information of drugs that are obtainable in many public databases. Machine learning is becoming an increasingly relevant tool for these three types of approaches [7,8,9,10,11]. Machine learning approaches [12] convert data concerning drugs and targets into features and apply confirmed drug–target interactions as labels for supervised training prediction models that can be used to predict interactions between new drugs and new targets.

Chemical genomics techniques [13] have been successful in drug discovery and retargeting in recent years. Although high-throughput screening and other bioassays have become possible, experimental methods for identifying drug–target interactions still require improvement in both throughput and practical application. Thus, developing effective non-experimental methods is imperative to deduce drug–target interactions in a cost- and resource-effective manner. Various computational models have been developed to solve the problem of drug–target prediction, and these models can be classified into different categories, such as machine-learning-based methods, network-based methods, and others [14]. Among these methods, machine-learning-based methods have attracted much attention due to their accurate prediction results.

Generally, machine learning techniques are used to predict drug–target interactions based on the chemical and biological properties of drugs and targets. Methods of machine-learning-based prediction can be divided into the following steps: First, the raw data of drugs and targets are preprocessed. Second, a suitable set of machine learning algorithms is employed to train the model with the preprocessed data. Third, the trained model is utilized to make predictions and generate new data. Machine learning was applied to predict drug–target interactions for the first time in 2007 [15] in a study that established a support vector machine (SVM) model by combining amino acid sequence, chemical structure, and mass spectrometry data. This success of this approach subsequently inspired many later studies.

Machine learning algorithms for drug target prediction are becoming increasingly popular. Classical machine learning methods, such as gradient enhancement [16], have been highly successful in prediction performance. Since the advent of deep learning technology, methods based on deep learning have been widely applied to DTI prediction. These methods have shown strong performance due to their ability to capture complex nonlinear molecular patterns. Luo et al. [17] proposed a method called DTINet that focuses on learning the characterization of low-dimensional feature vectors to project drug space onto target space optimally. OzuRk et al. [18] introduced DeepDTA based on SMILES for learning molecular characterization of target amino acid sequences and used a convolutional neural network to predict drug affinity. The DeepConV-DTI model proposed by Lee et al. [19] collected large-scale DTI datasets from different DTI databases, overcame the inaccuracy caused by DeepDTA’s performance evaluation on the same dataset, and optimized the convolutional neural network model to achieve better performance. For DTI prediction, graph neural networks were also used. GraphDTA, proposed by Nguyen et al. [20], translates SMILES strings into corresponding molecular graphs to represent drugs and targets and uses a graph neural network to predict drug affinity to each target. Moon et al. [21] proposed a new physical information map neural network called PIGNet, which provides more accurate and reliable drug–target interaction prediction results by combining physical principles and deep learning technology. Zhao et al. [22] proposed an improved graph representation learning method, iGRLDTI, for predicting drug–target interactions in heterogeneous bioinformation networks. Despite these efforts, most machine learning models still have limitations. In most cases, unlabeled biomedical data cannot be fully used for model training. Furthermore, the interpretability of models based on machine learning in DTI prediction requires improvement.

The interactive inference network (IIN) represents a novel neural network architecture designed to tackle Natural Language Inference (NLI) tasks. It notably enhances the performance of NLI tasks, particularly in managing complex semantic relations. This development is highly significant for advancing natural language processing technology and its applications, especially in scenarios that demand precise comprehension and inference of natural language data. Yichen Gong et al. [23] present an example of IIN architecture, Densely Interactive Inference Network (DIIN), which shows the most advanced performance on large-scale NLI corpora and similar corpora.

The present study adapted the architecture of DIIN and applied an interactive inference network for drug–target interaction (INDTI). The model learns the features of drug and target sequences at the embedding and encoding layers, simulates the interaction process through the interaction layer, extracts the neighborhood interaction relations via convolutional neural networks at the feature extraction layer, and finally outputs the prediction result. We applied the Explainable Substructure Partition Fingerprint (ESPF) algorithm [24] to segment drug and target sub-sequences in the embedding layer to use unlabeled biomedical data rationally. Furthermore, we used Morgan and PubChem molecular fingerprints for drug embedding as a supplement to obtain more characteristic information. Convolutional neural network autoencoders were used to encode sequences of molecules in order to capture the features of various functional groups of drugs and targets. Self-attention autoencoders were used to encode the sub-sequences of molecules in order to capture the correlation features between functional groups. The interaction layer simulates the interaction process. By visualizing the interaction space, we were better able to understand the drug–target interaction. INDTI achieves a high level of interaction predictability while simultaneously retaining the interpretability of drug–target interaction prediction.

## 2. Results

### 2.1. Performance Comparison between INDTI and Other Models

The embedding and coding layers of our model are highly extensible due to their modular design. It is possible to obtain different prediction effects and experimental significance by selecting different embedding methods and autoencoders. In Table 1, we compare the following methods of embedding and encoding and their effects on the interactive inference network model.

Our model was compared with the following DTI tools. Descriptions of each tool are provided below. In order to achieve the best results, the parameters used at baseline were as similar as possible to those in the original publication.

- DeepDTI [25] incorporated deep belief networks (DBNs) for DTI prediction. The combination of ECFP2, ECFP4, and ECFP6 was used as the drug input, and a protein sequence composition (PSC) was used as the target input. A PSC is composed of an amino acid composition (AAC), a dipeptide composition (DC), and a tripeptide composition (TC), which are, respectively, the frequencies of one, two, and three amino acids.
- DeepConv-DTI [19] used a CNN to extract local features of various lengths in amino acid sequences of targets and ECFP4 fingerprints of drugs and then input the combined feature vectors into the full connectivity layer to achieve prediction results.
- DeepDTA [18] employed a CNN to analyze SMILES strings and target amino acid sequences. In order to obtain the prediction results, the combined vectors were input to the full connection layer. A Sigmoid activation function was attached to its full connection layer for the DeepDTA task to be suitable for the binary prediction task in this publication.

We used standard performance measures for classification tasks, such as accuracy, precision, recall, and specificity. In addition, the ROC area was also used to measure a model’s generalization performance. The area of PR and F1 scores were used to measure precision and recall. Matthew’s correlation coefficients (MCCs) combine correlation coefficients between actual and predicted samples.

**Figure 1:**
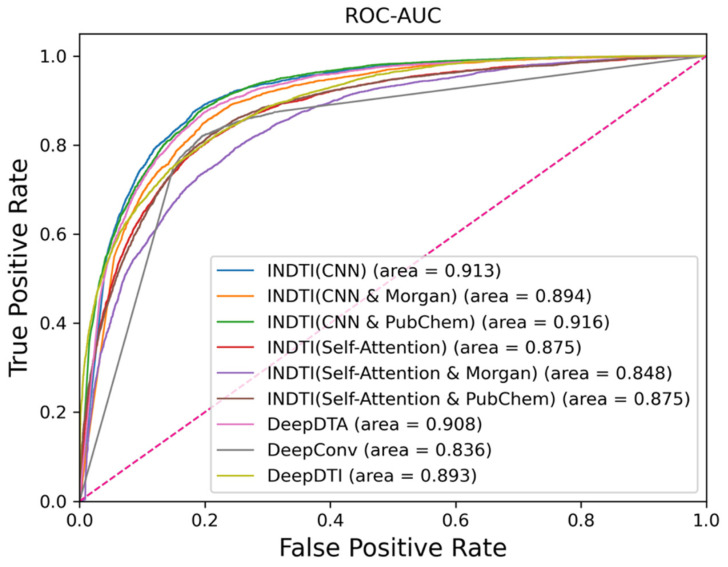
ROC curves of INDTI with different combinations of input and encoder and comparison to other algorithms. The solid lines indicate different algorithms, and the dashed line indicates random selection. The values in parentheses represent the area under the curve (AUC), which is a metric used to evaluate the performance of classifiers. The different combinations of modules used in INDTI are also listed in parentheses.

We compared our model with a baseline model under the same datasets, and the results are shown in Table 2. Figure 1 illustrates the ROC curves.

The INDTI model based on CNN extraction and combined with PubChem fingerprint extraction has the best performance, with an accuracy of 0.828, precision of 0.528, F1 score of 0.658, MCC of 0.584, and ROC-AUC of 0.916. According to the above evaluation metrics, it ranks first among all models. Its specificity is 0.818, ranking second. Although DeepDTI has the highest recall, its precision is only 0.378.

The PubChem fingerprint has the best performance in comparing the INDTI models combined with the CNN autoencoder. In contrast, the Morgan fingerprint has the worst effect, with low evaluation metrics. We observed that the INDTI model based on the self-attention autoencoder with three modes possessed distinct advantages. Feature extraction with SMILES sequences provided the highest accuracy, precision, specificity, and ROC-AUC levels. MCC and F1 scores were the highest when combined with Morgan’s fingerprint pattern. The recall and ROC-AUC were the highest when combined with PubChem’s fingerprint pattern. The CNN was excellent at capturing short-term memory. The molecular embedding was encoded using the CNN autoencoder, which was more suited to capturing the properties of each functional group of the molecule. Self-attention autoencoders can capture long-term memories and are able to capture associations between sub-sequences. When comparing the two autoencoders, the CNN has a better effect. The results suggest that drug–target interaction prediction may focus more on the sequence than the sub-sequence correlation.

The comparison results of the drug molecular fingerprint supplement are as follows: In the model using the CNN autoencoder, the effect of the model combined with PubChem is significantly better than that of the model combined with Morgan. In contrast, the self-attention-based autoencoder mode is the opposite, and Morgan’s mode performs better. This may be related to the method of fingerprint generation. PubChem records the presence of a substructure or a feature bit by bit. The Morgan representation of molecular structure is based on extending connectivity. Morgan is more suitable for extracting the sub-sequence correlation features based on the self-attention autoencoder. PubChem is more suitable for extracting sequence features based on the CNN autoencoder.

Compared to the other algorithms, the INDTI algorithm has higher accuracy, precision, specificity, F1 score, and MCC value in all INDTI modes than the DEEPConV-DTI and DeepDTI algorithms. The INDTI algorithm performs better in two models when compared with DeepDTA, a model based on CNN training, which uses only SMILES sequences to extract features, and the model based on CNN training that uses PubChem fingerprints. Thus, the INDTI algorithm displayed advantages over the other three algorithms used for comparison.

### 2.2. Robustness

In order to test the INDTI model’s ability to predict targets from different protein families, and to ensure that the model is not biased toward one specific protein family, we selected the model with the best performance in INDTI, which is based on CNN autoencoding combined with PubChem fingerprints. Five of the most common types of targets were examined: enzymes, ion channels, GPCRs, catalytic receptors, and nuclear receptors. To map all targets in the dataset by protein family, the GtoPdb database was used.

**Figure 2:**
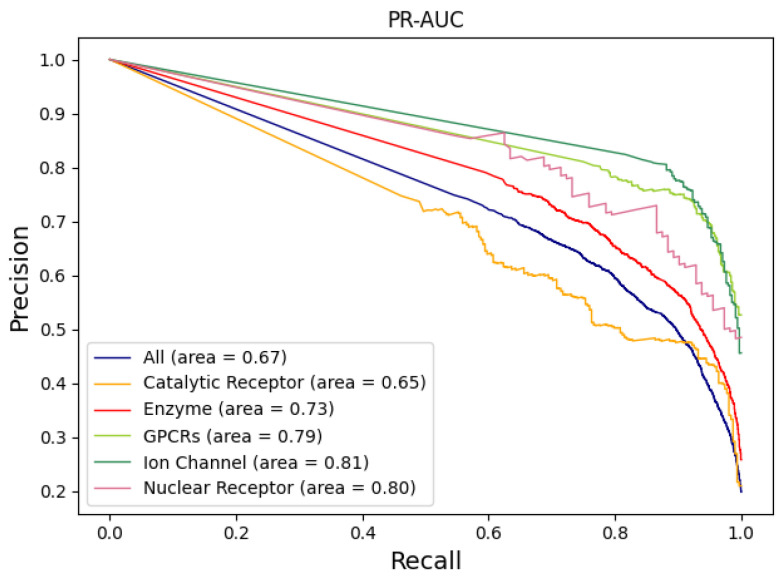
PR-AUC of different protein families. The solid line indicates different protein families. The values in parentheses represent the area under the precision–recall (PR) curve, which is a typical way to summarize a model’s overall performance.

**Figure 3:**
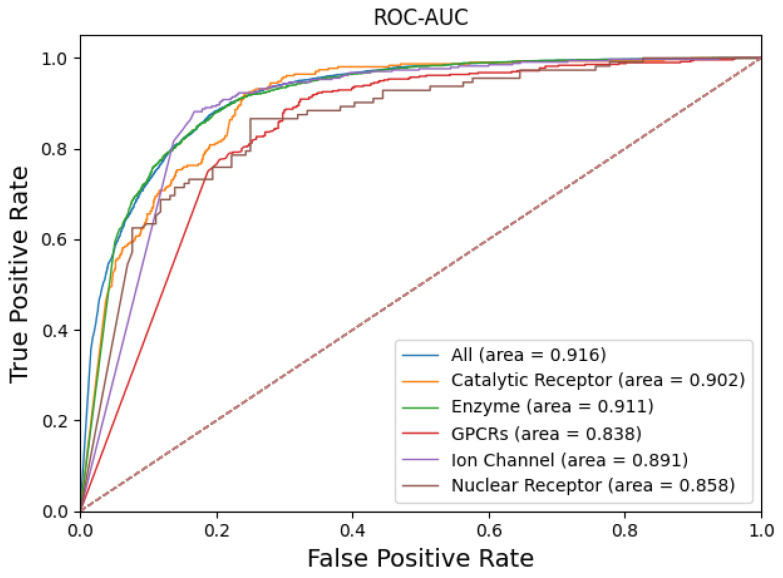
ROC-AUC of different protein families. The solid line indicates different protein families, and the dashed line indicates random selection. The values in parentheses represent the area under the curve (AUC).

This study examined 6844 pairs of enzyme–drug interactions, 1008 pairs of GPCR–drug interactions, 827 pairs of ion channel–drug interactions, 1637 pairs of catalytic receptor–drug interactions, and 271 pairs of nuclear receptor–drug interactions. PR-AUC is shown in Figure 2. ROC-AUC is shown in Figure 3. Table 3 shows the results of the evaluation metrics.

When it comes to MCC and ROC, most protein families individually are less effective than the whole protein family as a whole. The nuclear receptor (0.470) had the lowest MCC value of all protein families, which was 20% lower. The GPCR protein family had the lowest ROC value (0.838), 8% lower than the overall effect of the entire protein family. In other evaluation metrics, enzymes, GPCRs, ion channels, and nuclear receptors all scored higher on precision, recall, F1 score, and PR-AUC than the whole protein family. These results together suggest that with most of the protein families examined, INDTI displayed robust properties.

### 2.3. Interpretability

In drug–target interaction prediction, most models connect the feature vectors of drugs and targets. Dot products are used in the interaction layer of the INDTI model to simulate drug–target interactions, improving the algorithm’s performance and making interaction prediction more interpretable. A higher interaction value indicates a potential interaction between the drug’s substructure and target, which is crucial to the final interaction prediction. When using sub-sequence embedding, the encoded representations of drugs and targets can produce an interaction representation matrix between multiple drug sub-sequences and target sub-sequences through the interaction layer. We stack these matrices to obtain a single interaction representation matrix, which represents the interaction representation of drug and target sub-sequences.

**Figure 4:**
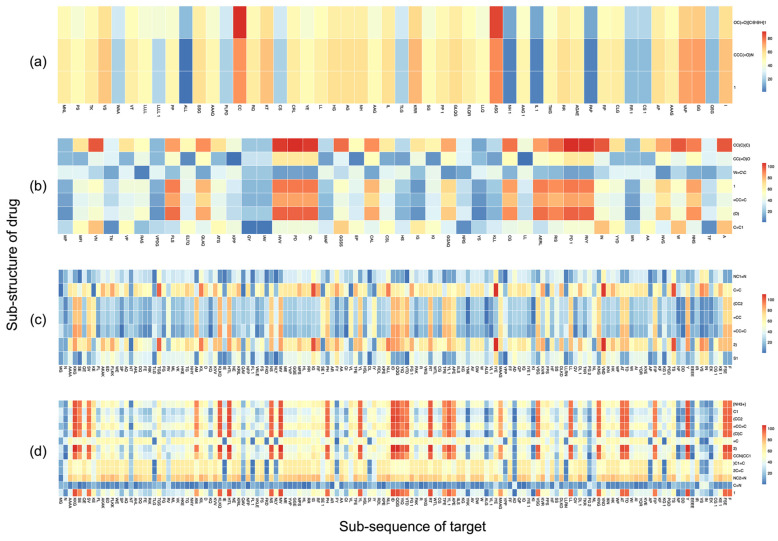
Drug–target interaction space diagram. (**a**) Pidolic acid interacts with Orexin. (**b**) 4-hydroxybenzaldehyde O-(3,3-dimethylbutanoyl)OXIME interacts with macrophage migration inhibitor factor. (**c**) 5-benzyl-1,3-thiazol-2-amine interacts with camp dependent protein kinase inhibitors. (**d**) 4-(4-chlorobenzyl)-1-(7H-Pyrrolo[2,3-D]Pyrimidin-4-yl)piperidin-4-aminium interacts with camp dependent protein kinase inhibitors. The different colors in the key represent the intensity of the absolute value of interaction value which was extracted from the hidden representation of the interaction layer.

When visualizing interaction maps, it is possible to observe which substructures are interconnected directly. For example, heat maps can be used to visualize the interaction, with the horizontal axis representing the substructures decomposed by the target amino acid sequence and the vertical axis representing the substructures decomposed by the drug SMILES string. In the interaction map, the corresponding colors of the color bar are mapped to the absolute value of the interactive value, and the absolute value distribution of each interaction map determines the value range. The higher the interaction value, the redder the color, representing a more significant potential contribution of each substructure to the interaction. The lower the interaction value, the bluer the color, and the lower the potential contribution of the substructure to the interaction. The INDTI model was run on four pairs of interacting drugs and targets, and the resulting interaction diagram is shown in Figure 4.

In predicting the interaction between Pidolic acid and Orexin (O43612), sub-sequence CC and ASG of Orexin showed prominent interaction with Pidolic acid. There was a potential interaction between the substructure 1=CC=C(O) of 4-HydroxybenzaldehydeO-(3,3-dimethylbutanoyl)OXIME and the sub-sequence HVVPDQL, CG, and AERLRISPDRVY of macrophage migration inhibitors (P14174). The sub-sequence (CC2=CC=CC=C) of 5-benzyl-1,3-thiazol-2-amine has an interaction activation relationship with most of the molecular sequences of camp-dependent protein kinase inhibitors (P17612). The other sub-sequences of camp-dependent protein kinase inhibitors have a high interaction value with the substructure of 4-(4-chlorobenzyl)-1-(7h-Pyrrolo[2,3-D]Pyrimidin-4-yl)Piperidin-4-aminium.

The heat maps are presented differently depending on how each pair of drugs and targets interact at the interaction layer. Drug sub-sequences can interact with certain target sub-sequences. In some instances, the entire drug sequence interacts with part of a target sub-sequence. Through the visualization of interaction maps, INDTI can provide support for understanding model prediction and illuminate the inner workings of drug and target mutual prediction models to a greater extent. It can provide as much information on drug–target interactions as possible to compare predicted results.

### 2.4. Prediction and Validation of Alzheimer’s Disease-Related Targets

In this study, we predicted and validated Alzheimer’s disease-related targets. The test drugs used were 11,913 approved, investigational, and other drugs obtained from DrugBank. A total of 11,297 drugs were available, excluding those for which no SMILES strings were available.

In order to ensure the reliability of validation results, all targets related to Alzheimer’s disease were excluded from the training and validation sets. Table 4 shows Alzheimer’s disease-related targets obtained from the Alzforum database (https://www.alzforum.org/alzpedia (accessed on 9 September 2023)) and related literature [26].

**Figure 5:**
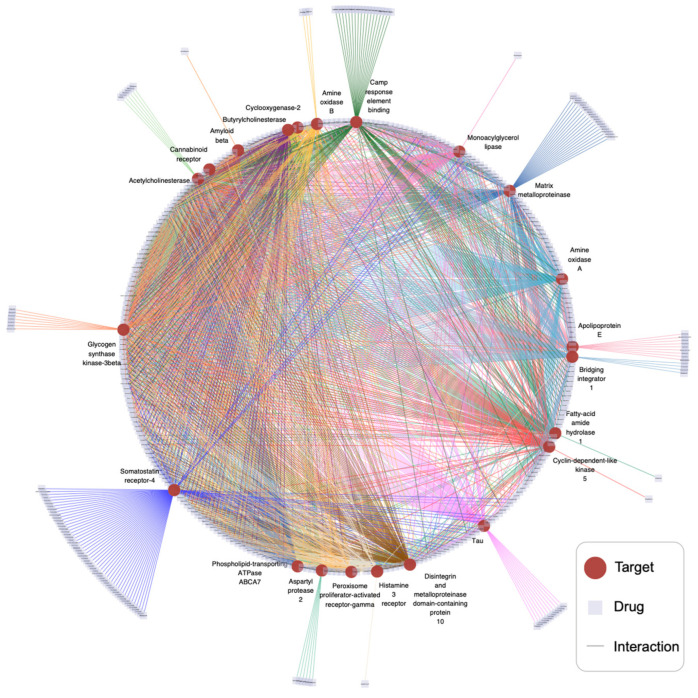
Prediction of Alzheimer’s disease-related drug–target interactions. The red dots in the interaction network diagram represent the 22 Alzheimer’s-related targets in Table 4. Each light purple square represents a drug, and the edge represents an interaction between the drug and the target. The edge emitted by each target is given a different color.

We predicted interactions with the 22 Alzheimer’s-related targets shown in Table 4. The predicted drugs that could interact with each target were ranked according to their probability of interaction. The top 100 drugs were chosen to construct the interaction diagram of the drug targets associated with Alzheimer’s disease (Figure 5). The connecting edge between Somatostatin receptor-4 and the drug is purple, and the connecting edge between Amine oxidase A and the drug is blue. In observing the target drug interaction network, it becomes clear that there are many drugs with cross-interactions among the top 100 drugs predicted by 22 targets and only a few drugs connected to one target. There may be a high degree of overlap between drugs targeting the same disease-related targets.

Amyloid-β polypeptide is produced by the intramembrane proteolysis of Amyloid precursor protein (APP). Mutations that cause inherited Alzheimer’s disease tend to increase the total Amyloid-β level or the ratio of Amyloid-β42 to Amyloid-β40 [27]. The formation of senile plaques in the brains of Alzheimer’s disease patients is caused primarily by the neurotoxicity and vascular toxicity of Amyloid-β [28]. Based upon this rationale, the target Amyloid-β was input into the INDTI model to form interaction pairs with 11,297 drugs. Of these, 1534 of the 11,297 predicted interaction pairs were predicted to be able to interact, and 9763 were predicted to be unable to interact. Twenty-three drugs interacting with Amyloid-β were obtained from the DrugBank database as verification to predict Amyloid-β interaction. SMILES sequences were not obtained for Aducanumab, CAD106, Mito-4509, and Gantenerumab. Among the other 19 drugs interacting with Amyloid-β, only one drug, Aluminum phosphate, was incorrectly predicted, while the other 18 interactions were correctly predicted. For the prediction result of 19 drugs interacting with Amyloid-β, refer to Supplementary Table S1.

Another key protein in Alzheimer’s disease, Tau, has been the subject of research since its discovery in 1975. It is not completely clear how Tau protein contributes to Alzheimer’s disease [29]. Nonetheless, tangles and deposits of hyperphosphorylated tau protein are commonly observed histopathologically in AD, frontotemporal dementia, and other neurodegenerative diseases [30]. The tau protein is thought to be altered by Amyloid-β, but the relationship between the two is unclear [31]. A Tau target was input into the INDTI model to form interaction pairs with 11,297 drugs, of which 2504 groups were predicted to interact, and the remaining 8793 groups were predicted not to interact. Through the DrugBank database, we identified five drugs that interact with Tau: Paclitaxel, Docetaxel, Astemizole, Lansoprazole, and Flortaucipir F-18. Paclitaxel, Docetaxel, Astemizole, and Flortaucipir F-18 were among the correct predictions. For the prediction result of five drugs interacting with Tau, refer to Supplementary Table S2.

A third protein underlying the etiology of Alzheimer’s Disease, Apolipoprotein E, is a secreted lipoprotein involved in cholesterol metabolism. It has three subtypes: ApoE2, ApoE3, and ApoE4. The APOE genotype is the most important genetic risk factor for Alzheimer’s disease. The ApoE4 allele has ranked first among genetic risk factors for Alzheimer’s disease since it was discovered in 1993 [32]. There is evidence that ApoE may influence the pathogenesis of AD through lipid homeostasis, synaptic conduction, and inflammatory damage to the blood–brain barrier. In addition, ApoE may contribute to the pathogenesis of AD by regulating lipid homeostasis, synaptic conduction, and inflammatory damage to the blood–brain barrier [33]. Five drugs interacted with Apolipoprotein E in the DrugBank database, namely Copper, Zinc, Zinc acetate, Zinc chloride, and Zinc sulfate, of which Copper and Zinc acetate were correctly predicted. For the prediction result of five drugs interacting with Apolipoprotein E, please refer to Supplementary Table S3.

In addition, we obtained eight drugs that interact with the target cyclin-dependent-like kinase 5 from the DrugBank database: Alsterpaullone, Alvocidib, Hymenialdisine, Indirubin-3′-monoxime, Olomoucine, SU9516, 6-phenyl[5H]PYRROLO[2,3-b]PYRAZINE, and Trilaciclib. Based on the model, their interaction with cyclin-dependent-like kinase 5 was verified. The results of Hymenialdisine and Trilaciclib were incorrect, while the others were correct. For the prediction result of eight drugs interacting with Cyclin-dependent-like kinase 5, refer to Supplementary Table S4.

From the BindingDB database, we also obtained non-interacting drug–target pairs associated with Alzheimer’s disease. We input them into the INDTI model for verification. Drug–target interactions in the BindingDB dataset are expressed as Kd values. Kd values greater than 30 indicate no interaction. The verification results are summarized in Table 5. The BindingDB database contained 15 non-interacting drug–target pairs associated with Alzheimer’s disease. The 15 pairs of genes are related to cyclin-dependent-like kinase 5 and Acetylcholinesterase, respectively. The prediction results revealed that 12 out of 15 drug–target pairs were correctly predicted, with an accuracy rate of 80%.

According to the above validation data, the drug–target interaction prediction model INDTI proposed here can effectively predict and provide reliable candidate screening for drug–target interactions.

## 3. Discussion

Here, we propose a prediction model, INDTI, based on the interactive inference network to address the problem of drug–target interaction prediction. The INDTI model has the characteristics of being multi-level and multi-stage. Its embedding layer and encoding layer may differ based on different characteristics and requirements. We decompose drug and target sequences using labeled datasets. The CNN autoencoder is used to encode short-term memories of molecular sequences. The self-attention autoencoder is used to encode long-term memories. INDTI components can be easily switched to achieve high compatibility and scalability. With INDTI’s interaction layer, it is possible to visualize the interaction space to better understand drug–target interactions. Thus, the internal working principle of the drug and target mutual prediction model can be explained to the greatest extent possible. Our analysis and comparison of the results use a variety of evaluation metrics, such as AUC and MCC, to compare INDTI with baselines. The comparison metrics we used demonstrate that INDTI is superior. At the same time, the experimental results also show that different embedding and coding methods will produce different results. This also suggests that we should consider selecting the most appropriate embedding and coding methods when dealing with specific tasks to improve prediction efficiency and reliability.

We tested the robustness of the INDTI model on different protein families. The results showed that the scores of PR-AUC and ROC-AUC of most protein families were higher than those of all protein families. The MCC value of the nuclear receptor protein family was the lowest, 20% lower than the values of all protein families. The ROC value of the GPCR protein family was the lowest, 8% lower than that of all protein families. In other evaluation indices, the scores of enzymes, GPCRs, ion channels, and nuclear receptors were also higher than those of all protein families. This indicates that INDTI is robust when used for different protein families. In terms of the single effect of most protein families, except for the nuclear receptor (the lowest MCC value is 0.470) and GPCR protein families (the lowest ROC value is 0.838), other protein families are not as good as all protein families in terms of MCC and ROC. This indicates that INDTI may need to be further optimized when dealing with some specific protein families.

In this research, we used the INDTI model to predict the drug–target interactions associated with Alzheimer’s disease. We first identified 22 targets associated with Alzheimer’s disease and then used the INDTI model to predict the interactions between these targets and 11,297 drugs. Among the predicted top 100 drugs, many drugs have cross-interactions; that is, they can interact with multiple targets. Only a few drugs are linked to a single target. This suggests that different drugs for the same disease may have highly overlapping targets. This result should help us to understand the drug action mechanism of Alzheimer’s disease and can also guide the research and development of new drugs to avoid repeated effects on the same target and reduce side effects. At the same time, it also shows that INDTI model can effectively predict drug–target interactions and provide valuable information for drug research and development. Overall, INDTI is an efficient and robust drug–target interaction prediction model.

## 4. Materials and Methods

### 4.1. Datasets

Our proposed model was evaluated on three datasets: BIOSNAP [34], BindingDB [35], and DAVIS [36]. We used the BIOSNAP database dominated by the MINER DTI dataset, which contained 4510 drugs and 2181 targets, as well as 13,836 drug–target interaction pairs from the DrugBank [37] database. DAVIS contains the Kd values for 68 drugs and 379 targets, and BindingDB contains the Kd values for 7165 drugs and 1254 targets. DTI pairs with Kd values lower than 30 can be considered positive.

**Figure 6:**
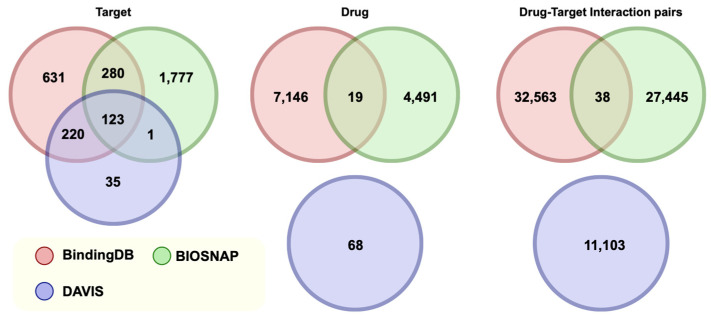
Venn diagram of relationships of DAVIS, BindingDB, and BIOSNAP datasets.

Different drugs, targets, and interactions are contained in the DAVIS, BindingDB, and BIOSNAP datasets. As shown in Figure 6, 403 targets overlap between the BindingDB and BIOSNAP datasets. A total of 343 targets overlap between the DAVIS dataset and the BindingDB dataset, 124 targets exist between the DAVIS and BIOSNAP datasets, and 123 targets overlap between all three datasets. The BindingDB and BIOSNAP datasets contain 19 drugs, while the BIOSNAP and DAVIS datasets do not have the same drugs. The BindingDB and BIOSNAP datasets both contained 38 drug–target interaction pairs, while the BIOSNAP and DAVIS datasets did not contain identical drug–target interaction pairs.

We combined data from three datasets to reduce the impact of differences between them. By stratifying the positive and negative samples of the datasets, we divided them into mutually exclusive subsets with a ratio of 7:1:2, which served as a training set, validation set, and test set, respectively. Table 6 shows the distribution.

### 4.2. Methods

**Figure 7:**
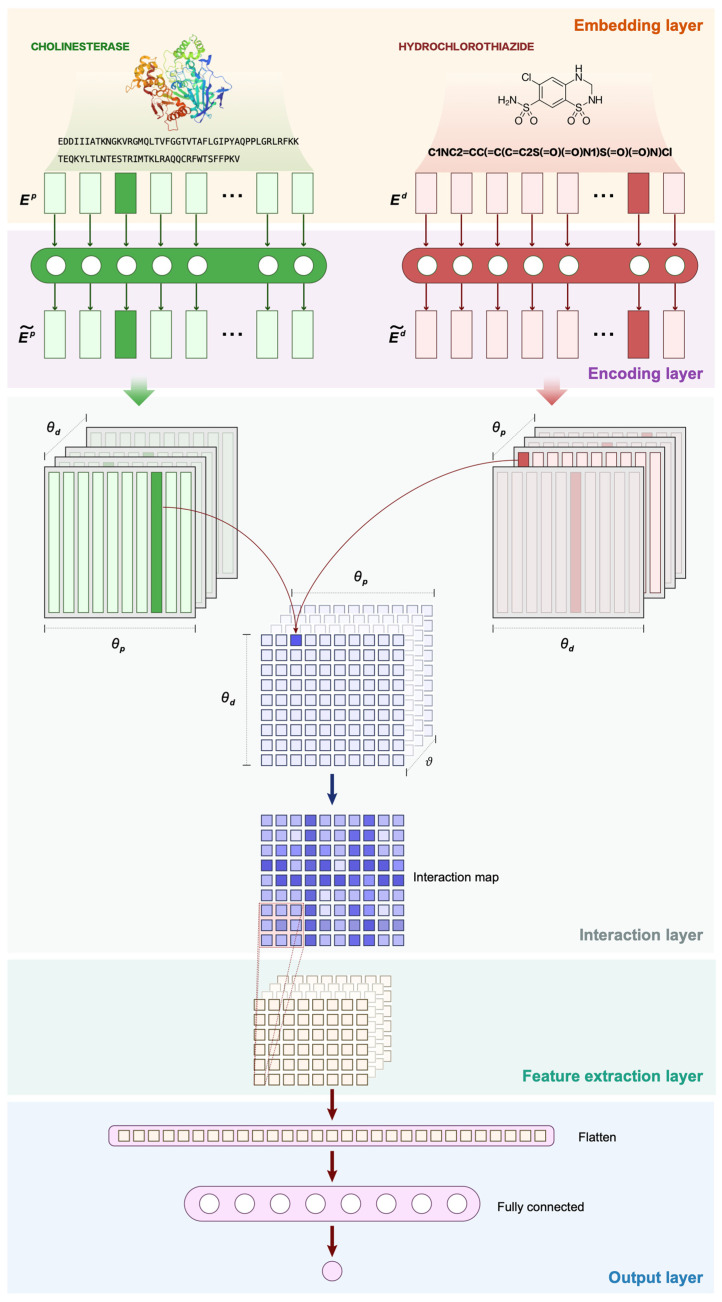
Model architecture of INDTI.

We propose an interactive inference network for drug–target interaction (INDTI) prediction algorithm. Based on an interactive inference network [23], INDTI has an embedding layer, an encoding layer, an interaction layer, a feature extraction layer, and an output layer. At the embedding layer, drug sequences and target molecules are embedded. The embedding of drugs and targets is encoded at the encoding layer. The interaction layer simulates drug–target interactions. In the feature extraction layer, the interaction features of the interaction matrix are extracted, and the prediction results are obtained. In general, when predicting the interaction between a target and a drug, most models connect the extracted features of both the drug and the target molecular sequences, feeding them into the prediction model. However, our model uses the dot product to generate a scalar indicating the interaction strength between a single target–drug minimum unit pair, which produces interpretable model interaction predictions. The proposed model framework is illustrated in Figure 7 with an example of cholinesterase (target) and hydrochlorothiazide (drug).

#### 4.2.1. Embedding Layer

Each drug and target sequence must be converted into sequence embedding at the embedding layer to encode the molecular sequence. We consider combining different embedding methods and encoders based on the encoder characteristics. In our study, we used three embedding methods: direct embedding, sub-sequence embedding, and fingerprint embedding.

Direct embedding. This is one of the most commonly used approaches for predicting drug–target interactions. Fixed letter symbols express these molecular descriptors, so they can be represented using fixed numbers. SMILES strings are concentrated in lengths of less than 100, and amino acid sequences in lengths of less than 1000. Thus, we set the maximum length of SMILES embeddings at 100 and the maximum length of amino acid sequence embeddings at 1000. The embeddings whose length is greater than the maximum length are intercepted, and those that are less than the maximum length are completed with 0.

Sub-sequence embedding. We decompose the complete molecular sequences of drugs and targets into medium-sized sub-sequences and treat each sub-sequence as an input whole. Explainable Substructure Partition Fingerprint (ESPF) [24] was used to extract drug and target substructures from the UniProt dataset [38] and the ChEMBL database [39]. The UniProt dataset contains 565,254 unique target sequences, while the ChEMBL database contains 2,105,464 drug SMILES strings. Table 7 shows that word collections (each denoted as V) of different sizes were derived using different frequency thresholds and data sources.

**Figure 8:**
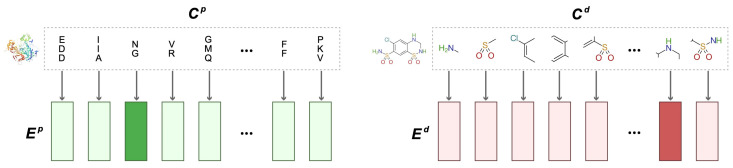
Molecular sub-sequence embedding process.

The original drug and target sequences are decomposed into k frequent sub-sequences $C=\{C_{1},\ldots ,C_{k}\}$, each of $C_{i}$ comes from the word collection V. The sequence decomposed into frequent sub-sequences is transformed into a bit vector, where each bit corresponds to an item in the found set of sub-sequences. SMILES and amino acid sequences are decomposed into multiple sub-sequences and are embedded according to the collection V. The process is illustrated in Figure 8.

The maximum length of drug $\theta_{d}$ is 50, and the maximum length of target $\theta_{p}$ is 545. The target sequence $C_{p}$ and drug sequence $C_{d}$ decomposed into substructures are transformed into two matrices, $M^{p}\in R^{k\times \theta_{p}}$ and $M^{d}\in R^{l\times \theta_{d}}$, where k and l are the size of the word collection V. Rows $M_{i}^{p}$ and $M_{j}^{d}$ of the matrix represent the one-hot encoding of the i-th sub-sequence of the target and the j-th sub-sequence of the drug, respectively. The embedding $E^{p}$ and $E^{d}$ of each target and drug are generated by the content embedding lookup matrix, and the generation rules are as follows:

(1) $$E^{p}=W_{content}^{p}M^{p}$$

(2) $$E^{d}=W_{content}^{d}M^{d}$$

where $W_{content}^{p}\in R^{\vartheta \times k}$ and $W_{content}^{d}\in R^{\vartheta \times l}$ are lookup matrices.

Fingerprint embedding. The two methods we use to embed fingerprints are fingerprint direct embedding and fingerprint marker embedding. Since a molecular fingerprint $C_{fp}$ consists of binary digits (0 and 1), it is directly readable. Furthermore, since the molecular fingerprint is long and the information density is sparse, it can be compressed by marker embedding. We identify all $C_{i}^{fp}$ of values 1 in molecular fingerprint $C^{fp}=\{C_{1}^{fp},C_{2}^{fp},\ldots ,C_{n}^{fp}\}$. The position i corresponding to the marker $C_{i}^{fp}$ is extracted by the original fingerprint order. Next, the embedded is generated by the extraction order.

#### 4.2.2. Encoding Layer

The encoding layer encodes the drug and target embedding by extracting features from the context information. Through the encoded embedding, a vector can be obtained that is more appropriate. Our study adopted three types of encoding methods in the encoding layer.

Multilayer Perceptron (MLP) autoencoder. Fingerprint embedding $E_{d}^{fp}$ is simple, context-free, and nonlinear. By using the MLP autoencoder, we encode it with a molecular fingerprint directly generated by a drug and input it into the autoencoder. The MLP autoencoder superimposed three layers of fully connected neural networks, and the hidden layers used 1024, 256, and 64 neurons, respectively. Each layer of the network proceeds by multiplying the input vector $E_{d}^{fp}$ by the connection weight $W_{d}^{fp}$ of the hidden cell to obtain the current neuron’s output, and each cell’s output passes through a nonlinear activation function. After the three-layer neural network, the fingerprint encoding $\tilde{E_{d}^{fp}}$ is finally obtained:

(3) $$Z_{d}^{fp}=E_{d}^{fp}W_{d}^{fp}$$

(4) $$\tilde{E_{d}^{fp}}=ReLU\left(Z_{d}^{fp}\right)$$

CNN autoencoder. SMILES and amino acid sequences are embedded for up to 1000 units. Encoding can be performed using fully connected neural networks. However, a fully connected neural network forms connections with all upper neurons, and each unit has its own weight. It is easy to overfit and inconsistent with the nature of drug and target interactions based on functional blocks. Convolutional neural networks connect upper and lower neurons directly through the convolution kernel, and the units in the same convolution kernel share parameters. In this way, the local characteristics of molecules can be extracted, reducing the processing time while preserving useful information.

As an autoencoder, we use a three-layer one-dimensional convolutional neural network. The CNN autoencoder is overlaid with three one-dimensional convolutional neural networks, and convolution kernels of different sizes are used for each layer of drug and target. The drug encoder uses 4, 6, and 8 convolution kernels, while the target encoder uses 4, 8, and 12. Upon completing each convolutional layer, a pooling layer and a full connection layer are superimposed, and the encoding of the drug $\tilde{E^{d}}$ and target $\tilde{E^{p}}$ is obtained.

Self-attention autoencoder. The embedding length is relatively short due to molecular sub-sequence embedding. Autoencoders based on self-attention mechanisms can capture chemical semantics and context correlation between drug and target substructures.

The sub-sequence embedding of drugs and targets contains only content information. In order to make use of the context information of the molecular sequence, location information needs to be added. When integrating sequence information and content embedding, a self-attention autoencoder incorporates positional encoding. The positional encoding $E_{{pos}_{i}}^{p}$ and $E_{{pos}_{j}}^{d}$ for drugs and targets can be generated by a position lookup matrix:

(5) $$E_{{pos}_{i}}^{P}=W_{pos}^{p}l_{i}^{p}$$

(6) $$E_{{pos}_{j}}^{d}=W_{pos}^{d}l_{j}^{d}$$

where $l_{i}^{p}\in R^{\Theta_{p}}$ and $l_{j}^{d}\in R^{\Theta_{d}}$ are the one-hot encoding of the ith sub-sequence of the target and the jth sub-sequence corresponding to the drug, respectively, and $W_{pos}^{p}$ and $W_{pos}^{d}$ are the position lookup matrices automatically formed according to the maximum length and pre-set embedding length of the decomposed sub-sequence of the drug and target. The input embeddings $E_{i}^{p}$ and $E_{j}^{d}$ of the self-attention autoencoder are obtained by adding the content embeddings and position embeddings.

The generated embeddings are fed into the self-attention autoencoder, which consists of N identical layers. Each layer has two sublayers. It is a multihead self-attention encoder in the first pass. Through different linear transformations, the multihead self-attention encoder combines different results of attention. We used the 12-head self-attention mechanism:

(7) $${head}_{i}=attention\left({QW}_{i}^{Q},{KW}_{i}^{K},{VW}_{i}^{V}\right)$$

(8) $$MultiHead\left(Q,K,V\right)=Concat\left({head}_{1},\ldots ,{head}_{h}\right)$$

Afterward, the feed-forward neural network feeds the attention layer’s results. A normalization layer follows both the self-attention layer and the feed-forward neural network layer. We obtained the encodings of drugs and targets $\tilde{E^{p}}$ and $\tilde{E^{d}}$:

(9) $$\tilde{E^{p}}=Attention\left(E^{p}\right)$$

(10) $$\tilde{E^{d}}=Concat\left(Attention\left(E^{d}\right),Attention\left(E_{d}^{fp}\right)\right)$$

#### 4.2.3. Drug–Target Interaction Prediction

In this module, the interaction between a drug and a target is simulated after the drug and target are encoded. This module has three layers: (a) the interaction layer simulates the interaction between drug and target; (b) the convolution layer extracts neighborhood sequence interactions; (c) the linear transformation of the whole connection layer is applied, and the prediction results are normalized.

In order to simulate the interaction between the pair of drugs and targets, for each target sub-sequence i and each drug sub-sequence *j*, the interaction value is generated:

(11) $$I_{i,j}=F\left(\tilde{E_{i}^{p}},\tilde{E_{j}^{d}}\right)$$

where *F* is the aggregation function used to measure the interaction between drug–target pairs; we used the dot product. The matrix *I* considers the interaction of a single minimum unit of the target and the drug. Using the dot product as an aggregation function, a single scalar can be generated to measure the strength of the interaction between a single target–drug minimum unit pair.

A two-dimensional interaction matrix *I* is output by the interaction layer, and features are extracted by the downstream layer. The higher the interaction matrix value, the higher the chance of DTI interaction. When a pair of interaction elements interact, their dot products will be higher at their corresponding pair positions in the interaction table *I*. Visualizing the interaction table helps us understand which units contribute to the final result. As a result, the internal working principles of the drug and target mutual prediction model can be elucidated as much as possible, which can provide strong support for biological interpretation.

Adjacent units may trigger interactions between target and drug sequences. It is therefore necessary to simulate the interactions between adjacent regions. Feature extraction is achieved using a convolutional neural network on interaction matrix *I*. Convolution kernels can capture adjacent interactions. The convolution layer output matrix *O* is obtained by

(12) $$O=CNN\left(I\right)$$

The output matrix *O* is tiled through the full connection layer, and the distribution features are mapped to the sample label space. The prediction result *L* is obtained:

(13) $$P=\sigma \left(W_{o}FLATTEN\left(O\right)+b_{o}\right)$$

(14) $$L=Y\log\left(P\right)+\left(1-Y\right)\log\left(1-P\right)$$

where $\sigma \left(a\right)=\frac{1}{1+\exp(-a)}$; $W_{o}$ and $b_{o}$ represent the weight matrix and offset of the full connection layer, respectively; and *Y* represents the real label of the drug–target interaction pair.

#### 4.2.4. Input Representation

The mapping between molecular structure and molecular properties needs to be expressed in molecular representations that are expressive enough for machines to understand. Molecular descriptors are used to describe the physical and chemical properties of molecules with normative symbols. Molecular descriptors convert physical and chemical information in molecular symbols into useful numerical indicators or the results of standard experiments. DeepDTA, DeepPurpose, and other models [18,40,41] learn characterization from SMILES strings of drugs and amino acid sequences of targets.

By using SMILES and amino acid sequences for drugs and targets, we can reduce the loss of native information. We also used molecular fingerprints to complement some chemical and substructural characteristics divided by chemical structure. Molecular fingerprinting is a degree of simplification or abstraction created to make comparisons and calculations between molecules easier. Two molecular fingerprints we use are Morgan and PubChem. The Morgan fingerprint [42], also known as the Extended Connectivity Fingerprint (ECFP), is a circular topology fingerprint based on the Morgan algorithm. The PubChem fingerprint [43] consists of 881 bits and is encoded with the multi-hot encoding mode, where each bit indicates whether the fingerprint has specific substructures and features.

In this study, SMILES and amino acid sequences were used as the characterization inputs of drugs and targets. Morgan and PubChem fingerprints were used as supplement inputs to extract the molecular characteristics of drugs.

## Figures and Tables

**Table 1:** The combinations of the selected embedding method and encoder.

Drug Input	Target Input	Encoder
SMILES	Amino acid sequences	CNN
SMILES + Morgan		CNN, MLP
SMILES + PubChem		CNN, MLP
SMILES		Self-Attention
SMILES + Morgan		
SMILES + PubChem		

**Table 2:** Comparison results.

Model		Accuracy	Precision	Recall	Specificity	F1	MCC
INDTI	CNN	0.820	0.514	0.862	0.810	0.644	0.566
	Morgan and CNN	0.813	0.504	0.839	0.807	0.629	0.545
	PubChem and CNN	0.828	0.528	0.874	0.818	0.658	0.584
	Self-Attention	0.812	0.502	0.780	0.819	0.611	0.515
	Morgan and Self-Attention	0.811	0.500	0.796	0.814	0.614	0.521
	PubChem and Self-Attention	0.800	0.482	0.816	0.800	0.606	0.513
DeepDTA		0.810	0.512	0.853	0.806	0.630	0.564
DeepConv-DTI		0.789	0.469	0.834	0.779	0.600	0.508
DeepDTI		0.700	0.378	0.897	0.780	0.533	0.441

**Table 3:** Test results with different protein families.

Protein Families	Accuracy	Precision	Recall	F1	MCC
All	0.828	0.528	0.874	0.658	0.584
Catalytic Receptor	0.794	0.480	0.870	0.619	0.534
Enzyme	0.820	0.588	0.880	0.704	0.606
GPCRs	0.744	0.679	0.958	0.794	0.532
Ion Channel	0.755	0.649	0.967	0.777	0.581
Nuclear Receptor	0.695	0.600	0.928	0.727	0.470

**Table 4:** Alzheimer’s disease-related targets.

Target Gene	Target
AChE	Acetylcholinesterase
BChE	Butyrylcholinesterase
App	Amyloid-β
BACE1	Aspartyl protease 2
SSTR4	Somatostatin receptor-4
MGLL	Monoacylglycerol lipase
MAPT	Tau
GSK3B	Glycogen synthase kinase-3β
CDK5	Cyclin-dependent-like kinase 5
MAOA	Amine oxidase A
MAOB	Amine oxidase B
PTGS2	Cyclooxygenase-2
ApoE	Apolipoprotein E
PPARG	Peroxisome proliferator-activated receptor-γ
CREB	Camp response element binding
HRH3	Histamine 3 receptor
MMP	Matrix metalloproteinase
ABCA7	Phospholipid-transporting ATPase ABCA7
ADAM10	Disintegrin and metalloproteinase domain-containing protein 10
BIN1	Bridging integrator 1
CB	Cannabinoid receptor
FAAH	Fatty-acid amide hydrolase 1

**Table 5:** Validation of drug–target pairs that cannot interact.

Drug	Target	Kd	Prediction Result
1-[4-[(4-ethylpiperazin-1-yl)methyl]-3-(trifluoromethyl)phenyl]-3-[4-[6-(methylamino)pyrimidin-4-yl]oxyphenyl]urea	Cyclin-dependent-like kinase 5	1000	√
DORAMAPIMOD		2000	√
BMS-387032		740	√
2-(2-chlorophenyl)-5,7-dihydroxy-8-[(3S)-3-hydroxy-1-methyl-4-piperidyl]chromone		110	√
JNJ-7706621		240	√
SELICICLIB		1900	√
US8765747, 2		150	√
Carbacylamidophosphate, 1a	Acetylcholinesterase	206,000	√
Carbacylamidophosphate, 2a		230,000	√
Carbacylamidophosphate, 3a		1,100,000	×
Carbacylamidophosphate, 4a		554,000	√
Carbacylamidophosphate, 1b		239,000	√
Carbacylamidophosphate, 2b		3820	×
Carbacylamidophosphate, 3b		2,950,000	×
Carbacylamidophosphate, 4b		4,260,000	√

**Table 6:** Statistics of DAVIS, BindingDB, and BIOSNAP datasets.

Type	Drugs	Targets	Pos. Interactions	Neg. Interactions
Train Set	8485	2861	15,648	15,648
Validation Set	4376	2356	2236	9596
Test Set	6708	2740	4471	19,192

**Table 7:** The scale of sub-sequence set with different frequency thresholds.

Type	Datasets	Frequency	Scale of V
Drug (SMILES)	ChEMBL	100	23,531
		1500	2585
Target (amino acid sequence)	UniProt	500	16,692
		2000	4113

## Data Availability

The DAVIS datasets are from the website http://staff.cs.utu.fi/~aatapa/data/DrugTarget (accessed on 8 October 2022). The BindingDB datasets are from the website https://www.bindingdb.org/rwd/bind/chemsearch/marvin/Download.jsp (accessed on 8 October 2022). The BIOSNAP datasets are from the website https://snap.stanford.edu/biodata/datasets/10002/10002-ChG-Miner.html (accessed on 8 October 2022). GtoPdb is located on the website https://www.guidetopharmacology.org/download.jsp (accessed on 8 October 2022). Alzforum is located on the website https://www.alzforum.org/alzpedia (accessed on 9 September 2023). DrugBank is located on the website https://www.drugbank.com (accessed on 1 July 2023). ChEMBL is located on the website https://www.ebi.ac.uk/chembl/ (accessed on 1 July 2023). UniProt is located on the website https://www.uniprot.org/ (accessed on 1 July 2023). The source code for our INDTI model is publicly available and can be downloaded from the following website https://github.com/XiaoZheBrother/INDTI (accessed on 1 July 2023).

## Supplementary Materials

The following supporting information can be downloaded at: https://www.mdpi.com/article/10.3390/ijms25147753/s1.
